# Capital Impressions

**Published:** 1881-04

**Authors:** Ben. H. Pratt


### THE BISTOURY.

Vol. XVII. ELMIRA, N. Y., APRIL, 1881. No. 1

### Contributions.

### CAPITAL IMPRESSIONS.

BEN. H. PRATT.

Without hoping to tell anything new, so threadbare has the inauguration and its accompanying circumstance been written up by the irrepressible and exhaustive press correspondent, whose number is legion and whose versatility is fathomless; without even touching, much less trenching upon, events that have lately transpired at the national capital; I have been led to believe that there may be enough of interest attaching to impressions made upon the mind of a visitor to the great political center, to shade up, and perhaps tint, in a faint way, the outlines of the telegraphic and correspondential pictures which the newspapers have presented, and to give added vividity to the pleasure of the viewing.

It is an experience, this living, moving and having one's being for three or four consecutive days in a crowd. To be jostled, and elbowed, and pushed, and pressed, and trodden upon as to one's toes, and crushed as to one's hat, and thumped upon all sides, and punched in all possible places, and sworn at, and growled at, and laughed at, and badgered about until one becomes so used to it that he doesn't feel natural unless somebody is jostling, or elbowing, or pushing, or pressing, or tramping, or crushing, or thumping, or punching, or swearing, or growling, or laughing, or badgering—this is what it is to go to a presidential inauguration at Washington. Would I advise one never to try it? By no means. It is an experienee one may get never otherwise, and one should not die without having realized it. But I anticipate.

Perhaps it will be best to say that one is over the worst when the opproach to the "City of Magnificent Distances" is heralded. The pilgrim then at least forgets his badgerment in conjecture concerning that which he is about to enter upon. How did I find Washington? Very much as most people find that of which they have heard much and seen little—very different indeed from the preconceived notions thereof. New York, Philadelphia, Boston, Baltimore—these may oft times disappoint the stranger—Washington, never. With Sheba's queen he can say "behold the half was not told me!" Of a new capital of a young republic one may not expect much. Of American taste and occidental continentalism one stores up no grand notions. Of Yankee brag and Jonathanic bombast one is apt to be exceedingly incredulous. Art, architecture, the humanities—these, one is apt to say, are not indigenous to the soil, and of them one is tempted to cry bosh when some other tonguily prates thereof in connection with our puerile civilization. Hence the stranger to Washington imagines that he is about to visit a grandly avenued tract which a hopeful and enthusiastic nation expects, in the uncertain future, to so build up, adorn and dignify, that it shall worthily attain to a position among the world's capitals—a place in the list of cities to which shall sometime attach honorable mention among the traveled—a *gloria non est, sed in futuro.*

That's just where we miss it. Rome was—Washington is. Of its beauty, its surprising individuality, its thorough singularity in its freedom from distracting commerce and the groveling tendencies thereof, its wondrous growth in all that serves to enlighten, elevate, cultivate, edify and polish; these, as they impress themselves upon the visitor in Washington, can scarcely be touched, much less dwelt upon, unless one have unlimited space to scribble over, unlimited time to devote to it, and a monopoly of adjectivular privileges. So I must touch, now here, now yonder, flitting from wonder to wonder, staying nowhere and bounding every whither, for lo, it is Washington!

And how like it is to a great big play-house which children of a large growth, and with unlimited means at their command, have vowed to make perfect. It would seem that the only design in building and adorning Washington had been to make it beautiful without reference to utility—to cultivate its art and its architecture as one would cultivate a garden of rare exotics, merely to gratify the æsthetic within the cultivators; this and only this. With avenues too broad for commerce—with a street pave too beautifully even and fragile and nice for practical utility—with the grandest models of classic architecture as pabulum for the taste constructive—with so much to evoke latent art and yet detain it within the bounds of the art proprieties without discovering the cords that hold it there—with few obstacles to contend with in the way of natural impediment to such advancement as the grand whim may suggest—aided in every undertaking by the fascinating beck of impatient aspiration—Washington is destined to become a city before which even the most famous of European or oriental capitals shall yet bow in abject deference.

Beautiful at any time, Washington is peculiarly so during such a pageant as that which quadrennially gives it special and national prominence, the inaugural season. It is then that the mosaic of our nationality becomes most vividly impressed upon the visitor. The dialectic differences and provincial oddities are then brought under closest observation, and throng all about us. The long-haired, broad-collared, dark eyed, swarthy faced, suspenderless, tight-booted, semi-swaggering, tobacco spirting chap needs no label. "I am a Southron, sir," is too plainly stamped upon every lineament of his being to be mistaken. The big-boned, heavy-shod, muscular, yet lean, good natured, impudently familiar, clad but not dressed, everywhere-at-home-fellow, doesn't need to say that he's from the upper New England states. That off-hand, contemplative and yet inquireful man, who doesn't wait for an introduction, and at the second meeting slaps you on the back with a peculiarly accented "hello," need not be located, for he's a westerner. The spruce metropolitan New Yorker is recognized by his seeing nobody he doesn't know, as he plunges on in his all accomplishing way. That timid looking one, awkward, yet quietly attractive in his way, who wants to know everybody he doesn't know and seems half sad because he doesn't know everybody he meets; who peers into every face he passes as though he were looking for his long lost brother; he is the interior Pennsylvanian—can't mistake him. The antipodes of the nation, with a sprinkling of the world's denizens thrown in, are present and in contact with you, and the antipodal assembling is a jolly contemplation for the fellow who feels his nobodity in particular, and has no positiveness, for the nonce, in his composition.

Of course these are not what one is supposed to go to Washington to see, especially upon an inaugural occasion; and yet one might go there to see less interesting things than these. Often those things which are secondary in our preconceived notions of a place become primaries, and one is often tempted, and successfully, to forget his chief errand in the prominence which attaches to the unexpected. The surprises one falls into are wont to absorb one's wits and lead him into a happy forgetfulness of the fact that he had an object in going to Washington, and he brings himself up standing, all at once, with the consciousness that he owes to himself and duty sundry points of interest he wots of, and which he has solemnly promised himself. Prominent among points on his visiting list is the big dome that looms up conspicuously over the the capitol building, and which for miles before he reached the city—if it were by daylight—riveted his gaze and challenged his wonder. He starts in its direction, threading the possible loops in the vast throng that crams that magnificent thoroughfare, Pennsylvania Avenue, the most generous highway the republic boasts—jostled by the thronging multitudes of cosmopolitans —every step an ecstatic thrill of enjoyment—every glance a revelation of sublimity of some sort, architectural, articultural, decorational or other—every moment a sensation of unspeakable delight of some kind—until one finds himself ascending, by mosaic serpentines, the capitol hill, and thence by stair-flights to that pigeon-hole looking door-way at the western center, which, despite the architect's pronunciamento to the contrary, and to the protest of every stranger, Washingtonians insist shall be called the front of the greatest legislative structure on the continent, and—one is within the capitol at Washington. Thousands have described it, so we meander its labyrinthian corridors ponderingly, but may not encroach upon the domain of our predecessors. Through archways, between columns, by paintings and statues, up massive staircases, along tesselated pavements, on, around and up, up, up; winding and wondering and resting and looking and still pushing up, up, up, until the great concave of the false dome, with its emblematical figures, is reached, and one meets his first disappointment, for, viewed so close at hand, the figures are distorted, appear out of drawing, are gross and rough, for they are made to be viewed from the rotunda floor, one hundred and eighty feet below, from which point we may view it, returning, but now up more flights of narrow spirals, dizzy-looking perches, but constantly serving for ascent and descent—up, up, up, until the highest available step is taken, and an exit made to the little railing that overtops the dome, and heavens! what a panorama is spread out to view. Like the spokes of a mighty wheel, divergent from the capitol as a hub, run out the avenues—Pennsylvania, New Jersey, North and South Capitol, Delaware, Maryland, East Capitol—stretching away in the dim distance until lost in the perspective. From here one distinguishes the White House, State building, Treasury building, Smithsonian Institution, Washington Monument, the Botanical Garden at your feet, the Arsenal, Long Bridge, Arlington Heights beyond the silvery sheen of the broad Potomac, circles and squares, from which again radiate other avenues—that away off on the east front, from which diverge North Carolina, Massachusetts, Tennessee and Kentucky avenues—those circular openings whence emerge Connecticut, New Hampshire, Massachusetts, Rhode Island and Vermont avenues, while in interminable criss-cross appear Georgia, Virginia, Ohio, Louisiana, Missouri and Maine avenues—all these but a grand network interminably and bewilderingly cut by the rectagular web of north and south and east and west running streets, the former the alphabetical norths and souths, and the latter the numerical ditto, presenting such a mazy tangle that it would seem as if a lifetime were too short to unravel it. But a quarter of an hour familiarizes the spectator with their labyrinthian character sufficiently for the time being, and gives one a very fair though unsatisfactory general idea of the topography of the great town. The sublimity of the view from the dome of the capitol deeply impresses every one that takes it, and it repays the tedious climb a thousand fold.

The descent from the domic height finds at least one almost compulsory stopping place, and that is at the first landing below the pictured false dome of the rotunda. One may spend an hour leaning over the railing here and watching the people upon the floor of the rotunda. These look flattened out—squeezed down to a dumpiness that carries one in imagination to dwarfland. The men look like hats under which there are only legs. These legs swing like pendulums under the hats, as men move hither and yon across the floor. The ladies look like open parasols with feathers on their tips. This point of view is one hundred and eighty feet above the floor. But let us down and to the front.

The eastern front of the capitol is a study over which one could edifyingly spend weeks of scrutiny. This is the side opposite the present populous part of the city, but in time will really be what it is now only by courtesy, *the* front. The great length of the building gives it a squatty look. It is 750 feet long. But for the dome, about 300 feet in height, this squattiness would be unendurable. Yet the portico, when one gazes from its floor into its top, seems a vastness in itself. Its 24 pillars surmounted by a pediment of 80 feet span, are singularly impressive. Taking in the extension porticos in front of the Senate and House, with their corinthian elaboration, and one is almost overpowered with the effect. Greenough's colossal statue of Washington, though erected at a cost of $44,000; disappoints the beholder. It was not made to be viewed at point-blank range, and seems a monstrosity where it now is, on the ground in front of the great portico. On the steps, at the south end, Persico's "Discovery," and on the north end Greenough's "Civilization," are considered great, and cost $25,000; but they do not satisfy the art student. Persico's allegorical group occupying the portico, embraces the "Genius of America," an eagle at her feet, a shield, an altar; "Hope," with an anchor; "Justice" with the constitution; these in sandstone. "War" and "Peace" flank the main doorway, and above the bronze portal is a bust of Washington, crowned by Fame and Peace. The massive bronze doors are wonders. They tell the story of Columbus and his discovery, in enduring brass. Here is Columbus before the Salamancan Council; his departure from the convent to the Spanish Court; his audience with Ferdinand and Isabella; his start from Palos; his landing at San Salvador; his first encounter with the natives; his triumphal entry into Barcelona; the Discoverer in chains; his death. In niches are his contemporaries Perez, Cortez, Ojeda, Vespucci, Mendoza, Alexander VI, Isabella, Ferdinand, Charles VIII of France, Henry VII of England, John II of Portugal, Balboa, Pizarro and others of his time; then his historians; over all COLUMBUS. The doors cost $30,000.

But I am not within the capitol yet, and my limit is already exceeded. Like the preacher at his "tenthly," with his time up, I close, without even a threat that it's "to be continued in our next."

